# Facilitating mental health service use for older Chinese immigrants: a case study of community-based organizations

**DOI:** 10.1093/geroni/igaa057.3367

**Published:** 2020-12-16

**Authors:** Yuanyuan Hu, Qingwen Xu

**Affiliations:** New York University, New York, New York, United States

## Abstract

New York City has the largest older Chinese population of any city in the United States. Older Chinese adults in New York City often meet significant stress to fulfill their needs, and mental health issues are common among this population (Mui, 1996; Dong, 2012). Despite the high prevalence, Asian Americans have the lowest rates of mental health services use compared to other ethnic groups (Abe-Kim et al., 2007). Additional to wide disparities in mental health access, older immigrants experience additional factors that affect their decision making to use mental health services. Limited knowledge exists about community-based organizations facilitating mental health services use for this population. This study aimed to fill this gap by case study approach and conducted a qualitative analysis of data collected as part of a study that investigated the resilience of the Chinese communities in New York City in the context of aging and immigration. Data from five community-based organizations serving this population were examined, through reading agency history and program introduction, visiting agency location and observing its operation, and interviewing the agency staff and program directors. Data collected were integrated, synthesized, and analyzed. Findings represent organizational staff’s perceptions of the mental health issues among older Chinese immigrants, needs and accessibility of mental health services, and facilitation of access and utilization of services by screening, education and referral. The qualitative results address individual help-seeking behavior and pattern, organizational response to and coordination of mental health needs, and capacity building on the community level.

